# The Principles and Practice of Modern Otology

**Published:** 1911-04

**Authors:** 


**The Principles and Practice of Modern Otology.** By **John F. Barnhill,** M.D., Professor of Otology, Laryngology, and Rhinology, Indiana University School of Medicine; and **Ernest deW. Wales,** B.S., M.D., Clinical Professor of Otology, Laryngology, and Rhinology, Indiana University School of Medicine. Second edition revised. Octavo of 598 pages, with 305 original illustrations, many in colors. Philadelphia and London: W. B. Saunders Company. 1911. (Cloth, $5.50; half morocco, $7.00, net prices.)

Barnhill has issued a second edition of his book his aim being to modernize the subject of otology. Not many extensive changes from the former edition have been made. Among the most important are the following: the chapter on the examination of the functions of the ear has been entirely rewritten and includes the description and formula of a uniform system of tests accepted by the Eighth Otologic Congress at Budapest in 1909; a more extended statement regarding operative injury of the facial nerve; a description of the conservative radical mastoid operation commonly known as the Heath operation and several paragraphs relative to symptoms, pathology and surgical treatment of labyrinth suppuration.

Throughout the entire work on all suitable occasions the fact is stated that prevention of aural diseases is usually easy in early childhood, whereas benefit or cure as a result of treatment in later life is often impossible. This book will be of much benefit to the general practitioner as well as the specialist. J. A. R.

